# 665. Clinical and Financial Impact of Next Generation Sequencing (NGS) in addition to Conventional Microbiology Testing in our Urban Referral Health Center

**DOI:** 10.1093/ofid/ofab466.862

**Published:** 2021-12-04

**Authors:** Vikram Saini, Tariq Jaber, James D Como, Rasha Abdulmassih, Zaw Min, Nitin Bhanot

**Affiliations:** 1 Allegheny General Hospital, Pittsburgh, Pennsylvania; 2 Allegheny Health Network, Pittsburgh, Pennsylvania; 3 Infectious Disease, Allegheny General Hospital, pittsburgh, Pennsylvania

## Abstract

**Background:**

Clinical microbiology traditionally relies on culture methodology and serological testing, that have inherent limitations. Newer diagnostic techniques such as Next Generation Sequencing (NGS) have shown promise to improve microbial identification. In select scenarios, we send clinical specimens to reference laboratories for NGS testing in addition to current standard of care (SOC) diagnostics. We wanted to determine how this diagnostic approach has impacted patient care. We also wanted to review the financial burden through cost-benefit analysis for these ‘send-out’ tests.

**Methods:**

We performed a retrospective chart review of all cases over a 3-year period in which NGS was submitted. Data, including demographics, comorbidities, antimicrobial use, and diagnosis (by SOC and NGS) were gathered. We delineated how often there was concordance or discordance between SOC and NGS. We also obtained information on financial cost (direct and indirect) and turnaround time (TAT) for NGS results.

**Results:**

A total of 33 clinical specimens from 25 patients were sent for NGS. The majority of specimens comprised joint tissue/fluid, organ tissue and CSF.

Concordance occurred between SOC and NGS testing in 75.8% (25/33) of samples; of those, 88% excluded infection. NGS identified a pathogen in 20% (5/25) patients in which concomitant SOC testing was negative. A subsequent change in antimicrobial management occurred in 16% (4/25) of patients. The mean TAT was 14 days and average cost per specimen was &821.52 (range: &573-&1590).

Table 1. Pathogens identified by NGS with negative traditional microbiological test results

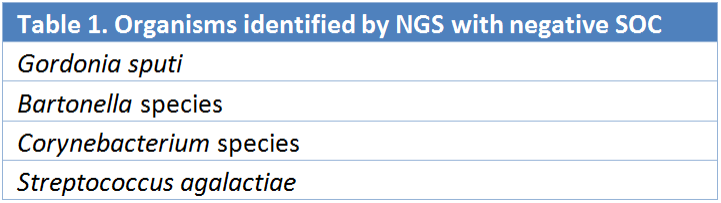

Figure 1. Distribution of specimen site (in %) sent for NGS

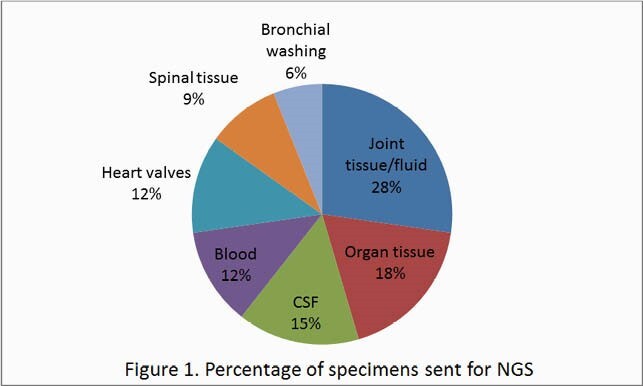

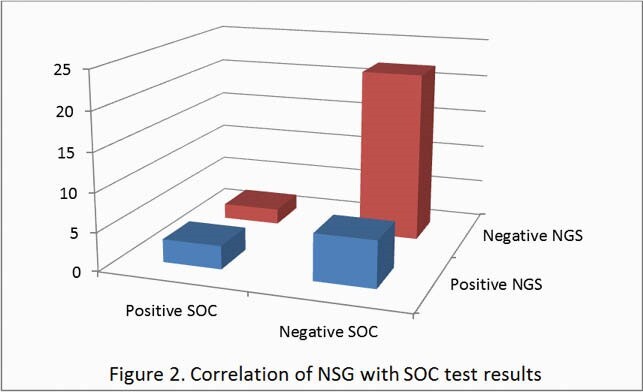

**Conclusion:**

NGS can provide additional diagnostic sensitivity in infectious diseases, which at our institution identified a new pathogen in 20% and a resultant treatment change in 16% of our patients. This testing may also allow physicians to reaffirm the absence of an infection diagnosis. A larger NGS testing population may reveal more significant benefits. While the attributable cost of NGS was substantial, it should be measured against the costs of administration of unnecessary antibiotics, inaccurate diagnosis, and adverse patient outcomes that may result from SOC testing alone. Considering its financial cost and extended TAT, in-house NGS testing may be warranted to facilitate a higher volume of testing.

**Disclosures:**

**All Authors**: No reported disclosures

